# Association Between Positive Mental Character and Humanistic Care Ability in Chinese Nursing Students in Changsha, China

**DOI:** 10.3389/fpsyg.2022.896415

**Published:** 2022-06-20

**Authors:** Lin Lai, Siqing Ding, Zhuqing Zhong, Ping Mao, Na Sun, Feng Zheng

**Affiliations:** ^1^Department of Cardiovascular Medicine, The Third Xiangya Hospital, Central South University, Changsha, China; ^2^Department of Nursing Teaching and Research Section, The Third Xiangya Hospital, Central South University, Changsha, China; ^3^Department of Nursing, The Third Xiangya Hospital, Central South University, Changsha, China

**Keywords:** positive psychology, humanistic care ability, positive mental characters, association, Chinese nursing students

## Abstract

**Aim:**

To investigate the status of positive mental characters and humanistic care ability among Chinese nursing students, and confirm the association between positive mental characters and humanistic care ability.

**Methods:**

A cross-sectional survey was conducted. Nine hundred eighty-one Chinese nursing students were recruited from hospitals and community healthcare services in Changsha, Hunan, China. Three different self-reported questionnaires were applied: The Demographic Characteristics Questionnaire, Humanistic care ability of Nursing Undergraduates Assessment Scale and Positive Mental Characters Scale for Chinese College Students. Pearson correlation analysis and multiple liner regression analysis were performed to analyze the association between positive mental character and humanistic care ability for Chinese nursing students.

**Results:**

The mean scores of nursing students' humanistic care ability and positive mental character were 125.94 ± 21.19, 233.18 ± 38.59, respectively. The Pearson correlation results showed that positive mental character (*r* = 0.655, *P* < 0.001) was significantly associated with humanistic care ability. Multiple liner regression analysis indicated that positive mental characters, four dimensions of courage, humanity, justice and transcendence in positive mental character, care from classmates were found to be independent predictors of humanistic care ability.

**Conclusion:**

Positive mental characters are important considerations in the development, implementation and evaluation of humanistic care ability interventions.

## Introduction

Nursing is a specialty that requires both the mastery and application of certain knowledge and skills, as well as the penetration of humanized nursing in clinical practice (Guo et al., 2018). Humanistic care is the ability to listen to the needs and desires of patients, understand patients' emotions, communicate with patients, and feel the value of life to develop therapeutic relationships (Rogers, 1981). Nursing humanistic care can help others maintain health in physiology, psychology, social culture and spirituality (Watson, 1979). At present, humanistic care has been applied in clinical nursing, nursing management, nursing education and so on (Xu and Tao, 2017). Humanistic care is mainly used in different departments such as pediatrics, oncology, geriatrics, and infectious diseases in clinical nursing in China. In terms of nursing management, researchers mostly formulate corresponding management strategies based on the elements of Human Care Theory, and create a human management environment for nurses to experience care (Zhang et al., 2019). In terms of nursing education, it is mainly to set up humanistic care courses for nursing students and clinical nurses or implement care practice through experiencing the care process (Zhang et al., 2019). The application of humanistic care improves the quality of clinical nursing, the satisfaction of patients and the professional recognition of nurses. However, due to the increasing workload of clinical nurses, the lack of nursing human resources, and the low quality of nurses, the implementation of humanistic care has been hindered to some extent. In order to solve these difficulties, in addition to reasonably increasing the staffing and reforming the management mode, it is more important to improve the humanistic care ability of nurses.

Whether nurses have good humanistic care ability depends on their humanistic quality education in school and clinical practice (Zhang et al., 2021). As a new force and an important reserve force in the nursing field, nursing students' humanistic care ability will directly impact the quality of nursing service and the overall quality of nursing teams in the future (Wang et al., 2020). In the training of nursing professionals, humanistic care education is the key means to transform humanistic ideas and humanistic knowledge into humanistic care ability (Zhuang et al., 2021). At present, the humanistic quality education system of nurses in China is mainly composed of schools, hospitals and nursing students. School classroom education is the main part, supplemented by the education of clinical teachers, which runs through the whole process from nursing students to nurses' career (Zhang et al., 2021). However, the cultivation of humanistic quality in China lacks systematic planning and strategic arrangement in the content of teaching materials, and the educational methods also lack scientificity and pertinence. The teaching hospitals did not closely combine practical teaching with humanistic quality education, and there was a phenomenon of emphasizing practice and neglecting humanities (Pang et al., 2018). Therefore, the cultivation of nursing students' humanistic care ability should be key content in nursing personnel training (Liu et al., 2017).

Studies have shown that the positive orientation of thoughts, emotions and behaviors of individuals is influenced by positive mental characters, which is reflected in the fact that individuals can maintain a positive and optimistic attitude when they are confused or in adversity, and are good at improving their self-quality when they are in good times, which has a positive effect on the caring behaviors of nursing students during their clinical practice (Park and Peterson, 2006; Celano et al., 2013; Kachel et al., 2021). Positive mental character was first mentioned by Meng et al. (2016) based on the researches from Peterson and Seligman (2004). Positive mental character is a series of psychological characteristics and their aggregate, which are accumulated in the deep heart of human civilization and national traditional virtues and keep pace with the times and embody the spirit of the times and core values. These psychological characters have distinct characteristics of enthusiasm, positive, initiative, progress, stability and construction. They include human inherent and acquired all the goodness and beauty, and constructive psychological characteristics and psychological forces, related in the cognitive style of positive, positive personality, positive relationships and so on. Positive mental characters provide positive energy for healthy and happy growth of individuals and harmonious development of society. Including 20 positive mental characters, such as creativity, bravery, kindness, fairness, modesty, gratitude and so on (Peterson and Seligman, 2004; Meng et al., 2016). With the development of positive psychology, interventions based on the theory of positive psychology have sprouted. These interventions are collectively referred to as positive psychological intervention (Sin and Lyubomirsky, 2009). Most of the positive psychological intervention strategies focus on positive characteristics, positive experience, positive thinking and positive relationship (Seligman and Csikszentmihalyi, 2000; Jiang and Tan, 2018; Tansey et al., 2018). The combination of classroom theory explanation, extracurricular activity practice and group psychological counseling can effectively improve the level of students' positive mental characters (Chen et al., 2021). Positive mental characters increase happiness, an ability to control one's emotions, thoughtful, logical thinking, gratitude and appreciation (Sansone and Sansone, 2010; Niemiec and Wedding, 2013; Morton, 2018). These characters are related to low rate of burnout, wellbeing and improvements in medical students' mental health (Mruk, 2008). Therefore, someone who possesses a more positive mental character are more likely to bring care, loving, understanding, feeling others' emotions and happiness to patients (Sansone and Sansone, 2010). However, the associations between positive mental characters and humanistic care ability have not been clearly explained.

Some studies have found that nursing students have some problems during the internship, such as lack of communication skills, lack of problem-solving ability, negative professional attitude and poor psychological adaptability. Humanistic care ability is the relevant factor affecting nurse-patient communication and problem-solving ability (Tian et al., 2019; Zhao et al., 2019). Therefore, it is important to improve the humanistic care ability of nursing students to complete the internship with high quality and improve their professional quality. While the importance of humanistic care in modern medicine is well recognized worldwide, the medical education sector faces a big challenge of helping the nursing students to develop the sense and ability of humanistic care. This study analyzed the current situation and the correlation between humanistic care ability and positive mental characters in Chinese nursing students in order to provide a reference for the implementation of future humanistic care interventions, and expected to improve the humanistic care ability from the perspective of positive psychology and the degree of professional recognition of nursing students as well as patients' satisfaction with nursing.

## Materials and Methods

### Design

Upon the approval of the Ethics Review Board of the Third Xiangya Hospital of Central South University (No: expedited review I 21064), a cross-sectional survey was conducted in Yuelu District, Changsha city, Hunan province, China, from June to July 2021.

### Participants

A stratified multi-stage cluster sampling was used to recruit the study participants. According to institution scale, specialties, technical facilities and academic orientation, all Chinese hospital and healthcare services are classified into tertiary hospitals, secondary hospitals and primary healthcare facilities. There are seven tertiary hospitals, 10 secondary hospitals and 15 primary healthcare facilities in Changsha's Yuelu District. Stratified cluster sampling was used with a stratified sampling ratio of 20% to randomly select three tertiary hospitals, two secondary hospitals and three community healthcare services in Changsha's Yuelu District. Nursing students at all levels (junior college nursing students, undergraduates and post-graduates) were invited to participate in the study. The following inclusion criteria were applied: (i) full-time nursing students; (ii) internship time greater than or equal to 1 month; and (iii) volunteered to participate in this study.

### Measures

#### Demographic Questionnaire

This was a researcher-developed questionnaire used to obtain information on sociodemographic variables, such as age, gender, registered residence, only-child family, education level, clinical practice time, care from classmates, experience in school societies, and experience in organizing activities.

#### Humanistic Care Ability of Nursing Undergraduates Assessment Scale (HCANU)

HCANU was compiled by Huang (2005). It contains 45 items that are distributed over the following eight dimensions: instillation of faith and wishes, healthcare education, formation of humanistic-altruistic values, scientific solutions for health problems, assistance with the fulfillment of essential needs, provision of a good environment, promotion of empathetic communication and help in overcoming difficulties. The response for each item was graded on a 5-point Likert scale: 0 = completely disagree to 4 = completely agree. The higher the score, the stronger the humanistic care ability. Among them, items 5, 6, 7, 20, 22, 29, 31, 33, 37, and 39 are reverse scores. A previous study confirmed the scale's acceptable reliability and validity, the Cronbach's α coefficient of the scale is 0.904, and the Cronbach's α coefficient of each dimension is 0.639–0.842 (Huang, 2005). The scale has a half-fold reliability of 0.925 and a content validity of 0.960 (Huang, 2005).

#### Positive Mental Characters Scale for Chinese College Students (PMCS-CCS)

The positive mental characters scale for Chinese college students was developed by Meng and Guan (2009) based on the theoretical framework of the Values in Action (VIA) and the questionnaire of the Values in Action Inventory of Strengths (VIA-IS) (Peterson and Seligman, 2004). It is a questionnaire with 62 items, which collects 20 positive mental characters distributed across the following six dimensions: wisdom and knowledge, courage, humanity, justice, temperance and transcendence (Meng and Guan, 2009). The response for each item was graded on a 5-point Likert scale: 1 = completely disagree to 5 = completely agree, with a higher score indicating a greater presence of the corresponding positive mental character. The Cronbach's α coefficient of the scale is 0.922, and the Cronbach's α coefficient of each dimension is 0.652–0.800 (Meng and Guan, 2009).

### Data Collection

Permission was granted by the director of each investigation unit before commencement. Counselors from the Nursing Teaching and Research Department at each institution gathered students for meetings on the first day of coming to hospital. At the end of these meetings, the researchers explained the purpose, study content and investigation procedures as well as the principle of study anonymity to participants before questionnaires were distributed. Written consent was obtained after nursing students agreed to participate in the study. Questionnaires were then distributed and completed by participants using paper-and-pencil method on the spot (it took ~15–20 min to complete the questionnaires). The questionnaires were collected immediately after completion, and checked for any missing information.

### Data Analysis

All of the collected data were entered into SPSS 23.0 (SPSS, Inc., Chicago, IL, USA to Armonk, NY: IBM Corp.) and analyzed. A *p*-value of <0.05 was considered statistically significant. Descriptive statistics were used to describe the nursing students' characteristics, and their level of humanistic care ability and positive mental character. Pearson correlation analysis was used to determine the correlation between positive mental character and humanistic care ability. Multiple liner regression analysis was conducted to analyze the association between the potential influential factors (i.e., social-demographic characteristics, positive mental character) and the dependent variable, humanistic care ability.

## Results

### General Characteristics of Surveyed Nursing Students

In this study, we initially identified 992 nursing students, among whom six refused to respond, and five were further deleted in data cleaning stage because of missing important variables. Therefore, a total of 981 valid responses were collected. The major characteristics of study subjects are shown in Table 1. There were 291 junior nursing students (29.7%), 591 undergraduates (60.2%) and 99 post-graduates (10.1%); 93 were male and 888 were female, with the mean age (SDs) 20.86 years (standard deviation (SD) = 2.17).

**Table 1:** Characteristics of nursing students, Changsha, Hunan, China (*N* = 981).

**Variable name**	**Group**	**No. of participants (*N* = 981)**	**Percentage (%)**
Gender	Male	93	9.5
	Female	888	90.5
Registered residence	Urban	634	64.6
	Countryside	347	35.4
Only-child family	Yes	210	21.5
	No	771	78.5
Education level	Junior nursing students	291	29.7
	Undergraduates	591	60.2
	Postgraduates	99	10.1
Clinical practice time	The 1st−3rd month	368	37.5
	The 4th−6th month	171	17.4
	The 7th−9th month	192	19.6
	The 10th−12th month	250	25.5
Care from classmates	More	36	3.7
	medium	343	35.0
	less	602	61.3
Experience in school	Yes	758	77.3
societies	No	223	22.7
Experience in organizing	Yes	592	60.3
activities	No	389	39.7

### Responses to Nursing Students' Humanistic Care Ability and Positive Mental Character

Details of humanistic care ability and positive mental character for 981 nursing students are displayed in Table 2. The mean scores for humanistic care ability and positive mental character were 125.94 ± 21.19, 233.18 ± 38.59, respectively. The mean scores for the humanistic care ability dimensions ranged from 8.57 to 26.67, with instillation of faith and wish as the highest score (26.67 ± 5.44), followed by healthcare education (21.13 ± 4.31). Provision of a good environment was the lowest score dimension (8.57 ± 2.57). For positive mental character, the dimensions ranged from the highest mean scores to the lowest mean scores, from wisdom and knowledge (44.18 ± 8.22), humanity (42.23 ± 6.96), courage (37.95 ± 6.56), transcendence (37.72 ± 6.60), temperance (37.33 ± 6.36) to justice (33.76 ± 5.86).

**Table 2:** Scores of humanistic care ability and positive mental characters for nursing students, Changsha, Hunan (*N* = 981).

**Scales and dimensions**	**Total items**	**Lowest score**	**Highest score**	**Mean ±SD**
**Humanistic care ability**	45	26.00	180.00	125.94 ± 21.19
Instillation of faith and wish	9	0.00	36.00	26.67 ± 5.44
Healthcare education	7	0.00	28.00	21.13 ± 4.31
Formation of humanistic-altruistic values	6	0.00	24.00	18.35 ± 3.80
Scientific solution of health problem	4	0.00	16.00	10.77 ± 2.61
Assistance with the gratification of essential needs	4	3.00	16.00	11.05 ± 2.18
Provision for good environment	5	0.00	16.00	8.57 ± 2.57
Promotion of sensibility communication	5	0.00	20.00	15.03 ± 3.13
Help for overcoming difficulty	5	6.00	17.00	10.39 ± 1.99
**Positive mental characters**	62	62.00	310.00	233.18 ± 38.59
Wisdom and knowledge	12	12.00	60.00	44.18 ± 8.22
Courage	11	10.00	50.00	37.95 ± 6.56
Humanity	10	11.00	55.00	42.23 ± 6.96
Justice	9	9.00	45.00	33.76 ± 5.86
Temperance	10	10.00	50.00	37.33 ± 6.36
Transcendence	10	10.00	50.00	37.72 ± 6.60

### Association Between Humanistic Care Ability and Positive Mental Character

Table 3 shows the results of the association between humanistic care ability and positive mental character. Positive mental character was positively correlated with humanistic care ability respectively (*r* = 0.656, *P* < 0.001). Each dimension of positive mental character was positively associated with humanistic care ability. In addition, positive mental character as a whole variable and each dimension within it were not found to be associated with the dimension of provision of a good environment in humanistic care ability.

**Table 3:** Correlation coefficients between positive mental characters and humanistic care ability of nursing students.

**Scales and dimensions**	**Humanistic care ability**	**Instillation of faith and wish**	**Healthcare education**	**Formation of humanistic-altruistic values**	**Scientific solution of health problem**	**Assistance with the gratification of essential needs**	**Provision for good environment**	**Promotion of sensibility communication**	**Help for overcoming difficulty**
Wisdom and knowledge	0.618^***^	0.600^***^	0.619^***^	0.525^***^	0.551^***^	0.478^***^	− 0.009	0.553^***^	0.137^*^
Courage	0.633^***^	0.623^***^	0.663^***^	0.582^***^	0.509^***^	0.506^***^	− 0.072	0.627^***^	0.125^*^
Humanity	0.655^***^	0.613^***^	0.644^***^	0.559^***^	0.558^***^	0.511^***^	− 0.018	0.603^***^	0.160^**^
Justice	0.642^***^	0.626^***^	0.626^***^	0.553^***^	0.553^***^	0.497^***^	− 0.005	0.608^***^	0.165^**^
Temperance	0.605^***^	0.594^***^	0.601^***^	0.542^***^	0.508^***^	0.451^***^	− 0.017	0.572^***^	0.120^**^
Transcendence	0.620^***^	0.604^***^	0.608^***^	0.529^***^	0.515^***^	0.469^***^	− 0.021	0.578^***^	0.157^**^
**Positive mental characters**	0.656^***^	0.641^***^	0.659^***^	0575^***^	0.560^***^	0.510^***^	−0.025	0.619^***^	0.151^**^

^*^p < 0.05, ^**^p < 0.01; ^***^p < 0.001.

### Humanistic Care Ability Associated Factors

Variables of nursing students' characteristics (i.e., age, gender, registered residence, only-child family, education level, clinical practice time, care from classmates, experience in school societies, and experience in organizing activities), positive mental characters and its six dimensions—wisdom and knowledge, courage, humanity, justice, temperance and transcendence, respectively were included in the multiple liner regression analysis as independent variables. Table 4 shows the results of multiple liner regression analysis of nursing students' humanistic care ability. Positive mental characters (*P* = 0.004), four dimensions of courage (*P* = 0.024), humanity (*P* = 0.019), justice (*P* = 0.004) and transcendence (*P* = 0.036) in positive mental character, care from classmates were found to be independent predictors of humanistic care ability. Individuals with more courage, humanity, justice and transcendence in positive mental characters were more likely to have better humanistic care ability. However, those with more care from classmates were more likely to have worse humanistic care ability. Among them, the independent variables of humanity has the greatest prediction power for humanistic care ability.

**Table 4:** Multiple liner regression analysis of determinants of humanistic care ability for nursing students, Changsha, Hunan, China (*N* = 981).

**Effect**	***B***	**SE**	**Beta**	***t***	***P***
Positive mental characters	0.209	0.073	0.381	2.878	0.004
Courage	0.757	0.333	0.269	2.275	0.024
Humanity	0.952	0.403	0.313	2.362	0.019
Justice	0.846	0.290	0.253	2.915	0.004
Transcendence	0.010	0.350	0.004	0.030	0.036
Care from classmates	−3.445	1.092	−0.134	−3.154	0.002

## Discussion

In this population-based cross-sectional study, we described the status of humanistic care ability and positive mental character, and explored possible influencing factors on humanistic care ability in Chinese nursing students. We found that, of the 981 participants, the mean (standard deviation) of humanistic care ability was 125.94 (21.19). Humanistic care ability scores in our study were higher than the 70.79–125.39 from previous studies conducted in China (Chen et al., 2015; Zhang et al., 2018b). This might be due to the evolution of the nursing profession in China, where humanistic care education has been receiving greater attention in recent years. Many universities have set up courses related to humanistic care, so nursing students' humanistic care abilities have improved. But compared with the results of Huang (126.74 ± 14.16), the level of humanistic care ability of nursing students in our study was lower, indicating that there was still room for further improvement (Huang et al., 2021).

We also found that among the humanistic care ability dimensions, the scores of instilling faith and wish, and health education, are higher than in the other dimensions, consistent with the findings of previous studies (Jiang et al., 2016; Zhang et al., 2018a). Instilling faith and hope is to encourage and support patients to establish confidence and make them full of good ideas and pursuit of curing diseases and restoring health (Huang, 2005). The reason for the high scores may be that the subjects of the study were nursing students. After starting clinical practice, most nursing students are full of enthusiasm and freshness for clinical nursing. When confronted with patients suffering from various illnesses for the first time, it is easy to stimulate their empathy and make them involuntarily care for patients, help patients alleviate pain and promote health. Delivering public health education is part of clinical nursing, and requires sufficient professional knowledge. Nursing students have taken most specialty nursing courses. The mastery of professional knowledge enhances nursing students' ability to provide health education to patients. Medical educators generally believe that students can acquire these core humanistic caring abilities through learning relevant courses, especially from observing senior role models (Logio et al., 2011; Branch et al., 2017). In our study, provision of a good environment dimension score was the lowest among the humanistic care ability dimensions. A possible reason may be that most nursing students have <1 year of clinical practice, so they have insufficient understanding of the clinical environment and patient needs. And due to the limitation of student identity, it is difficult to manage the patient environment well.

In the current study, positive mental characters were found to be positively correlated with humanistic care ability in nursing students, as were the dimensions of positive mental characters. It indicated that the more positive a nursing student's mental characters, the greater his or her humanistic care ability, which is consistent with the findings of Xu et al. (2019). Nursing students with high positive mental characters show self-confidence, optimism and strength in their studies and life. These positive characters will make them treat nursing service with a positive attitude. In terms of positive mental characters, humanity was the most strongly correlated with humanistic care ability (*r* = 0.655, *P* < 0.001), which was in congruence with the previous study by Xu et al. (2019).

Our study showed that nursing students' positive mental characters had been recognized as a factor affecting humanistic care ability according to multiple liner regression analysis. We also have found courage, humanity, justice and transcendence dimensions in positive mental characters were independent predictors associated with humanistic care ability. Positive mental characters are the psychological state of an individual in the process of growth and development (Bu et al., 2018), which can allow an individual to effectively respond to events and the environment. They can also improve work attitudes and increase an individual's love of their profession, which is the premise of humanistic patient care (Thompson et al., 2016; Houpy et al., 2017). Courage refers to students' authenticity, persistence and zest in 20 positive personality characteristics (Meng and Guan, 2009). Humanity refers to students' ability to feel love, love and kindness, and social intelligence (Meng and Guan, 2009). Justice refers to students' team spirit, fairness and leadership. Transcendence refers to appreciation of beauty and excellence, humor, faith and hope. The greater a person's courage, humanity, justice, and transcendence, the stronger their humanistic care ability (Kachel et al., 2021). It is possible that nursing students with more positive mental characters could provide services that better meet patients' needs, through effective patient communication, the observation of patient behavior and empathy with patients' emotions. It is suggested that raising the level of nursing students' positive mental characters could improve their humanistic care ability.

Positive mental characters are important for nursing students, and they also need to learn how to shape these positive characters. Xiong found that those who served as student cadres scored higher in positive mental characters such as courage, persistence and enthusiasm. Therefore, nursing students should be encouraged to run for student cadres and actively organize or participate in various school activities and social practice, so as to cultivate their spunk and perseverance. Good interpersonal communication ability is an essential quality in the implementation of humanistic care (Xiong, 2016; Tang, 2018). Through non-violent communication, self-confident expression, and social skill training, students can develop the positive mental character of humanity and build harmonious interpersonal relationship. Justice embodies leadership and cooperation. The group cooperative learning can promote students to help each other and improve together, which is of great benefit to cultivate students' cooperation, equality consciousness and character of justice. The essence of transcendence is the cultivation of faith. nursing educators should guide nursing students in establishing appropriate values for their profession and a correct outlook on life (Xiong, 2016). This will not only make it easier for students to achieve their ideal goals, but also to improve self-efficacy, enhance happiness and cultivate hope for the future, significantly improving their humanistic care ability (Lukat et al., 2016).

The authors assume that positive mental characters are not “set in stone” and the environment can also shape them (Peterson and Seligman, 2004; Ping, 2019; Wang et al., 2019). Educators should effectively learn from the ideas and propositions of positive psychology, and understand the role of positive mental character in humanistic care ability. In terms of professional courses, we should encourage the study of humanities and social sciences. By changing the teaching methods of the course, integrating positive psychology into it, and offer interdisciplinary courses when necessary, so as to cultivate the humanistic quality of nursing students (Wendy and Noël, 2018).

In this study, care from the classmates was reported to be the significant determinants by multiple liner regression analysis. The finding is not consistent with the result of previous researches, which found someone feeling more care and love from the people is also more likely to feedback care and love to others (Simmons and Cavanaugh, 1996; Li and Cheng, 2016). Maybe because there were only 3.7% nursing students who feeling more care from the classmates, 61.3% nursing students who feeling less care from the classmates in our study. Our population is unbalanced in terms of care from the classmates, it is also suggested that the caring atmosphere is absent in school. Educational environment determines the quality of education. A good educational environment, such as a loving school environment, plays a subtle role in the cultivation and development of nursing students' humanistic care ability (Huang, 2005). Nursing colleges should pay attention to the construction of humanistic care atmosphere and promote the exemplary role of teachers in caring for students. Encourage nursing students to be willing to communicate and share the happiness and frustration during the internship. Help each other and feel warmth, so that nursing students can fully feel and understand the meaning of care, and gradually form the awareness and ability of humanistic care.

### Study Strengths and Limitations

A few strengths of this study are as follows: the findings showed that positive mental characters are related to humanistic care ability, whereas few prior studies examined the association between them. The results of the study will raise educators' awareness of the impact of positive mental characters on students. In addition, based on the existing findings, researchers can develop targeted and operational intervention programs, and explore specific measures to improve the humanistic care ability of nursing students from the perspective of positive mental character.

There are some limitations in this study: first, this study was conducted in only one province of China, and does not represent the entire country, therefore further research should be conducted in an expanded sample size in expanded areas. Second, our population is unbalanced in terms of gender, with the ratio of male to female nursing students ~1–9.5. Additionally, this study was a cross-sectional survey. It was impossible to fully grasp the dynamic changes of the positive mental character and humanistic care ability of nursing students, and further exploration was needed.

## Conclusion

Humanistic care ability is one of the core abilities of clinical nurses. It is a new way to improve the ability of humanistic care from the perspective of positive psychology. Colleges and practice hospitals should reform the educational model, organically combine the training of positive mental characters with the teaching process. Establish a positive psychological intervention mechanism from four aspects: courage, humanity, justice and transcendence. In addition, we must pay attention to the emotional communication with students, so that students fell being cared for. These all contribute to the shaping of the positive mental characters of nursing students, so that they can be more proactively involved in clinical practice, thereby enhancing their humanistic care ability.

## Data Availability Statement

The raw data supporting the conclusions of this article will be made available by the authors, without undue reservation.

## Ethics Statement

The studies involving human participants were reviewed and approved by the Ethics Review Board of the Third Xiangya Hospital of Central South University. The patients/participants provided their written informed consent to participate in this study. Written informed consent was obtained from the individual(s) for the publication of any potentially identifiable images or data included in this article.

## Author Contributions

LL and FZ designed the study. LL, PM, NS, and FZ collected data and performed statistical analysis. LL drafted the paper. SD, ZZ, and FZ revised the paper. All authors have read and approved the final version of the manuscript.

## Conflict of Interest

The authors declare that the research was conducted in the absence of any commercial or financial relationships that could be construed as a potential conflict of interest.

## Publisher's Note

All claims expressed in this article are solely those of the authors and do not necessarily represent those of their affiliated organizations, or those of the publisher, the editors and the reviewers. Any product that may be evaluated in this article, or claim that may be made by its manufacturer, is not guaranteed or endorsed by the publisher.
